# Thermal Decomposition of Metacinnabar (β-HgS) during Monoethylene Glycol Regeneration in Natural Gas Processing

**DOI:** 10.1021/acs.energyfuels.5c00428

**Published:** 2025-04-14

**Authors:** Chengyi Hong, Xiaopeng Huang, Tzu-An Lee, Yuanhao Zhou, Jonas Wielinski, Marcus Mello, Raja Jadhav, Daniel Chinn, Evan S. Hatakeyama, Thomas Hoelen, Gregory V. Lowry

**Affiliations:** † Department of Civil and Environmental Engineering, Carnegie Mellon University, Pittsburgh, Pennsylvania 15213, United States; ‡ Chevron Technical Center, 100 Chevron Way, Richmond, California 94802, United States; § Chevron Oil, Products, and Gas, San Ramon, California 94583, United States

## Abstract

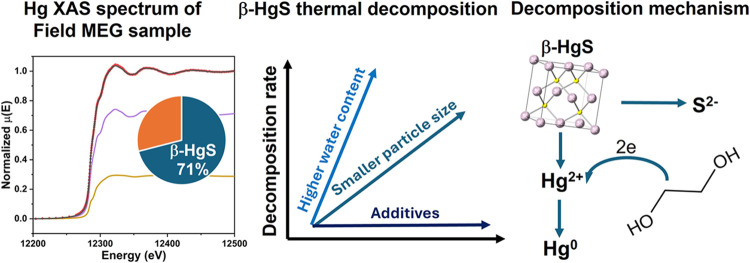

Elemental mercury and mercury (Hg)-bearing particles may be present in gas and condensate from specific geologic reservoirs and be coproduced with them. In this study, we found that over 70% of the Hg mass in field monoethylene glycol (MEG) is present as 100–200 nm particulate β-HgS, and it is therefore important to understand the decomposition behavior of β-HgS in MEG to determine the partitioning of mercury species in liquid natural gas (LNG) plants. Thermal decomposition studies in MEG and MEG-water solutions showed that β-HgS decomposition to elemental mercury started at around 100 °C, which is significantly lower than the 200 °C required for β-HgS decomposition in an inert gas. Density functional theory calculations supported the experimental observations that β-HgS has a lower decomposition temperature in solvents than its counterpart without a solvent because the solvent interactions decrease the Hg–S bond strength. Thermal decomposition studies at 130 °C showed that increased water content and decreased β-HgS particle size significantly increased the decomposition rate, while some common additives in field MEG did not have a significant effect. Experiment results suggest the decomposition pathway of β-HgS in MEG/water includes dissolution to form dissolved Hg(II) ions, followed by reduction to form elemental mercury by reaction with MEG. This study highlights the strong effect of solvent on the thermal decomposition mechanism of β-HgS, improving our understanding of the fate and species of Hg in petrochemical processing.

## 1 Introduction

Elemental mercury and mercury (Hg)-bearing particles can be present at trace levels in gas and condensate extracted from geologic reservoirs during oil and gas production.^1−3^ Proper management of these streams can prevent unwanted releases of Hg into the environment.^4,5^ We reported previously that particulate metacinnabar (β-HgS) is the most common Hg species in the majority of the petroleum value chain.^6^ Understanding the thermal decomposition and fate of β-HgS during oil and natural gas processing aids in the prevention of potential mercury releases to the environment and the potential damaging effects of elemental mercury on processing equipment.^7−9^

One process in natural gas extraction is inhibiting hydrate formation,^10−12^ which uses monoethylene glycol (MEG) as a hydrate inhibitor to absorb the water in extracted natural gas to enable compression or transport in pipelines. The used MEG with high water content, commonly referred to as rich MEG, is regenerated to lean MEG by distillation for reuse.^13^ In this regeneration process, the rich MEG is heated at 130–145 °C to boil off the water.^14,15^ Several studies have been conducted to understand the behavior of elemental mercury in these fluids; however, there is little work to understand the behavior of particulate mercury.^16−18^ Hg-bearing particles, such as β-HgS in natural gas and condensate fluids, can partition into the MEG and, therefore, are also subjected to heating in the regeneration process. Thermal decomposition of Hg-bearing particles to volatile elemental Hg has been reported in the regeneration process,^19^ which can escalate the risk of Hg damage to equipment or potential releases to the environment. The mechanism for the thermal decomposition of β-HgS in the MEG-water system at this relatively low temperature has not been reported.

The thermal decomposition of β-HgS in the gas phase has been studied. For instance, Wu et al. used Temperature-Programmed Decomposition Desorption Mass Spectrometry (TPDD-MS) to detect the release of elemental Hg from several mercury compounds at high temperature.^20^ In a helium atmosphere, dry β-HgS powder started to thermally decompose to elemental Hg at ∼200 °C. The reduction of Hg(II) coincided with the oxidation of S(-II) with the end products of Hg(0) and S_*x*_(0). β-HgS also can be decomposed into Hg(0) and S_2_ at 150 °C in hydrocarbons.^21^ These decomposition temperatures are higher than in the MEG regeneration process (130–145 °C) where generation of elemental Hg was detected.^15^ Thermal decomposition requires breaking the Hg–S bonds in β-HgS and is an endothermic reaction. The reaction medium (inert gas vs liquid solvent) can influence the electronic structure of minerals like β-HgS and their reaction products, affecting the thermodynamics parameters for its decomposition.^22,23^ In addition to the reaction fluid difference, the size distribution of β-HgS and coexisting constituents added to MEG or picked up from the geological fluids (e.g., chloride or bromide)^24^ also have the potential to influence the thermodynamic and kinetic parameters for the β-HgS thermal decomposition reaction. These variables in the MEG regeneration process were investigated to better understand the β-HgS decomposition in oil and gas processing.

Density functional theory (DFT) calculations can reveal how variations in the electronic and/or molecular structures can affect chemical reactions, such as the dissociation of Hg–S bonds of β-HgS. Cluster models of β-HgS can be used to obtain the electronic structures^25,26^ and vibrational analyses needed to predict the thermodynamic parameters of a chemical reaction (Gibbs energy (Δ*G*), enthalpy (Δ*H*), and entropy (Δ*S*)) as a function of the medium properties, temperature, or pressure. This approach can be used to calculate the lowest thermal decomposition temperature possible in either gas or solvents, such as MEG or MEG-water mixtures, to determine the effects of the different solvents on the energy of the reaction.

In the present study, we first characterized the size, elemental composition, and Hg speciation of Hg-bearing particles in MEG samples collected from a natural gas facility (field MEG) using single-particle inductively coupled plasma time-of-flight mass spectrometry (sp-ICP-TOFMS) and X-ray absorption spectroscopy (XAS). We then determined the thermal decomposition of synthesized β-HgS particles in MEG, water, and MEG-water solutions for different particle size distributions and in the presence of coexisting constituents in the solvents. The thermal decomposition of β-HgS in field MEG was also measured for comparison with the model systems. Finally, DFT was used to calculate the thermodynamic parameters of β-HgS decomposition (Δ*G*, Δ*H*, and Δ*S*) in the different solvents, and the thermal decomposition reaction mechanism was determined.

## 2 Materials and Methods

### 2.1 Synthesis of Metacinnabar (β-HgS) Particles

Monoethylene glycol (MEG) was purchased from Honeywell, and all other reagents were purchased from Sigma-Aldrich. Two field MEG samples (A and B) were collected from an MEG regeneration process in a natural gas processing facility. A simplified natural gas processing flow is shown in Figure S1. The β-HgS used in this study was synthesized using a sono-assisted precipitation method.^27^ Briefly, 2.4 g of Hg(Ac)_2_ and 0.75 g of thiourea (SC(NH_2_)_2_) were dissolved into 150 mL of deoxygenated water that had been purged by N_2_ gas for at least 45 min. Under continuous N_2_ bubbling, the solution was sonicated for 30 min to form dark-colored particles. The as-prepared particles were separated by centrifugation and washed by water and ethanol several times and dried at 60 °C overnight.

The morphology and elemental distribution of the synthesized β-HgS were determined by using a scanning electron microscope coupled with energy dispersive spectroscopy (SEM-EDS, FEI Quanta 600, US). The crystalline structure of β-HgS was confirmed using X-ray diffraction (XRD, Empyrean, Malvern Panalytical, U.K.) with nickel-filtered Cu Kα radiation generated at 45 kV and 40 mA.

### 2.2 sp-ICP-TOFMS Analysis for Hg-Bearing Particles in Field MEG

To characterize the size and elemental composition of individual Hg-bearing particles from field MEG samples, the particles were first collected on a 0.1 μm poly(vinylidene fluoride) (PVDF) membrane filter (Merck Millipore). Part of the filter cake was placed in 25 mL of water and sonicated for 30 min. After dilution, the particle suspensions (Sample A and B) were analyzed by sp-ICP-TOFMS (TOFWERK, Thun, Switzerland) as described elsewhere.^28^ The sp-ICP-TOFMS measurements were performed without reaction gas (no-gas mode) to obtain particle size distributions and again using dioxygen as a reaction gas (O_2_-reaction mode), which enabled the simultaneous detection of Hg (and any other metals) and sulfur (S) in individual particles simultaneously as previously described.^28^

### 2.3 Hg Speciation in Field MEG Samples by XAS

To determine the Hg speciation in the Hg-bearing particles, the filter cake of Sample A was analyzed by XAS at the Hg L_III_-edge (12284 eV) on BL 11–2, Stanford Synchrotron Radiation Facility (SSRL), as previously described.^6^ Due to the low Hg concentration in Sample A, the sample filter cake was directly sealed with Kapton tape without dilution. All measurements were conducted in liquid nitrogen at 77 K. With HgCl_2_ salt as a reference, the spectra of model compounds and Sample A were collected in transmission and fluorescence mode, respectively. The scans were conducted from 12,054 to 12,264 eV with a 10 eV increment, from 12,264 to 12,314 eV with a 0.35 eV increment, and then until 12.25k with a 0.05k increment. Nine model compounds were scanned, including cinnabar (α-HgS), metacinnabar (β-HgS), HgCl, HgCl_2_, Hg-thiosulfate, Hg-tetrathiolate (Hg(SR)_4_), Hg-cysteine, Hg-cystine (Hg(SR)_2_), and Hg-phenyl. The details of the model compounds are shown in Table S1. The data and linear combination fitting analysis (LCF) was performed in Athena v. 0.9.26. Both EXAFS and normalized XANES regions of Sample A were fitted using linear combinations of model compound spectra, with EXAFS being fit in *k*^2^-space over the range of *k* = 2–9 Å^–1^. During LCF, one compound with a major contribution was first identified and fitted to the sample spectrum. Then, additional compounds are added only if they lower the residuals by more than 10%.

### 2.4 Experimental Setup

**Figure 1:**
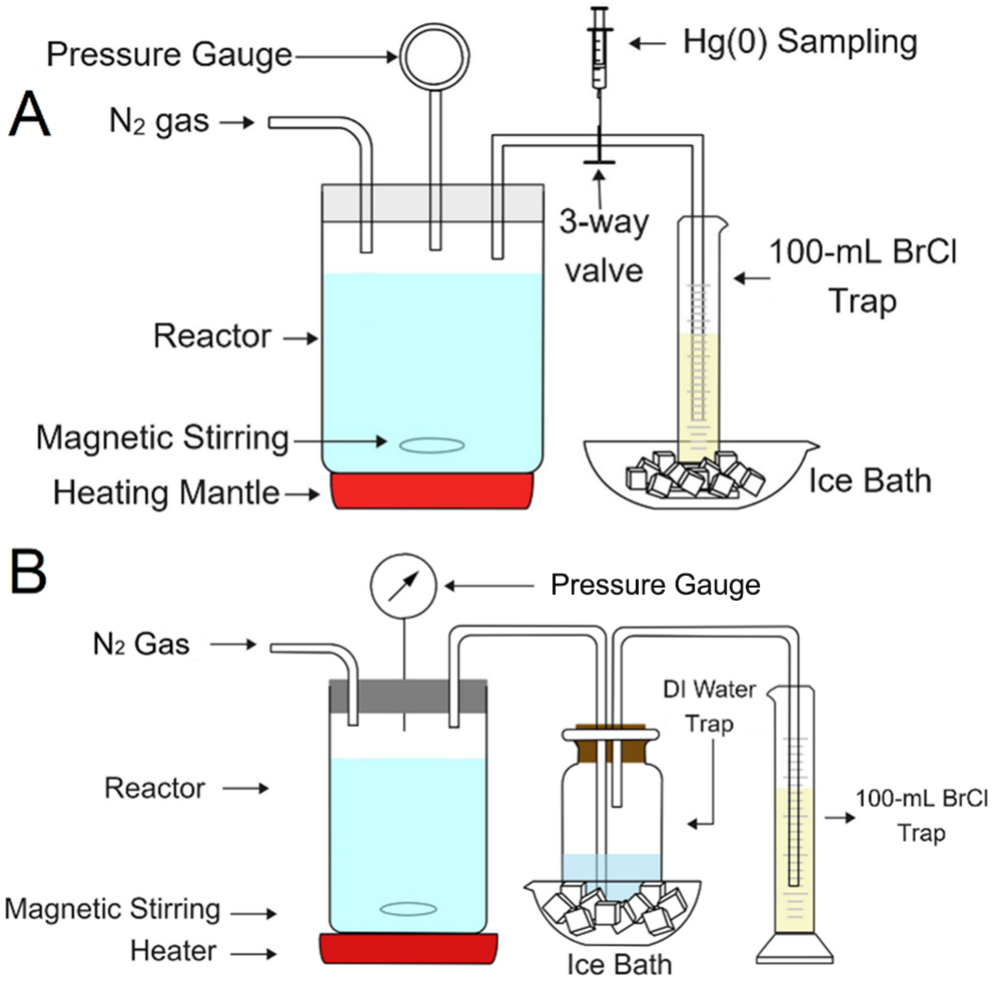
Schematic of the β-HgS thermal decomposition measurement system. (A) One-trap system with the reactor and BrCl trap. (B) Two-trap system with the reactor, water trap, and BrCl trap.

The thermal decomposition of β-HgS was measured in a heated reactor with a Hg trap system (Figure 1A). The stainless-steel reactor was customized by Xiamen Ollital Technology to operate at high temperature and pressure, up to 300 °C and 10 atm, respectively. To prepare β-HgS suspensions having different particle distributions for thermal decomposition, 1 mg/mL of a β-HgS dispersion was prepared by briefly sonicating the particles in water in a graduated cylinder. After settling for different amounts of time, the upper layer of the suspensions containing the smallest particles was collected, and the intensity-weighted average hydrodynamic diameter size distribution was measured in DI water by dynamic light scattering (DLS, Malvern, U.K.) using a 10 s acquisition time and refractive index of 2.90. Longer settling times result in smaller particle size distributions for use in the reactor.

For thermal decomposition measurements, 7 mL of the β-HgS suspension was added to 173 mL of MEG-water solution in the reactor (Figure 1A) to reach the final volume of 180 mL. Starting at room temperature and 300 rpm of magnetic stirring, the reactor was gradually heated to 130 °C, which replicated the operating temperature of the MEG regeneration process. The total heating time lasted for 150 min. The *t* = 0 time in all figures correlated to the time when *T* = 60 °C in the reactor (there was no β-HgS decomposition before 60 °C). Using N_2_ continuously flowing through the reactor headspace during operation, the produced gaseous elemental Hg and water vapor in the reactor outlet were collected in 100 mL of 0.002 M BrCl solution in a 250 mL graduated cylinder in an ice–water bath. At predetermined intervals, an aliquot was collected from the trap for Hg measurement and the solution volume was recorded for calculating a mass balance. Gas samples (5 mL) were also periodically collected directly at the reactor outlet and analyzed to qualitatively confirm the presence of elemental Hg in the reactor outlet gas stream. After thermal decomposition, residual β-HgS in the reactor was dissolved by aqua regia and BrCl. Then, the dissolved β-HgS was transferred to a 250 mL volumetric flask for measuring the total residual Hg in the reactor. The Hg mass balance was calculated by comparing the initial mass of β-HgS added with the Hg mass collected in the trap plus the residual β-HgS. The β-HgS decomposition for different particle size distributions, MEG-water ratios (0% MEG, 100% MEG, 60%:40% MEG:Water), and in the presence and absence of coexisting constituents (NaBr, CH_3_COONa or methyldiethanolamine (MDEA)) were conducted to explore the influence of these parameters on the thermal decomposition of β-HgS.

In the case of the field MEG sample, volatile organics in the reactor effluent consumed BrCl and lowered the trap efficiency for elemental Hg (Figure 1B). To overcome this, a gas washing bottle containing 100 mL of deionized water was placed between the reactor and the BrCl trap cylinder to trap VOCs emanating from the field MEG samples. Experiments used 180 mL of field MEG, and the heating and sampling methods using the BrCl trap were consistent with the methods for β-HgS decomposition. After the thermal decomposition was complete, the Hg(0) concentration in the water trap was also measured, and the final trap volume was recorded. In addition, the total Hg in the water trap and all residual β-HgS in the reactor were dissolved and oxidized by aqua regia and BrCl to obtain a full Hg mass balance.

To investigate the thermal decomposition mechanism, batch experiments were used to evaluate the reduction of dissolved Hg(II) (added as HgCl_2_) to elemental Hg in MEG solvents with different water contents. The synthesized β-HgS was also heated in a two-trap system (Figure 1B). The details of these experiments are described in Texts S1 and S2.

### 2.5 Hg Analysis Method

All Hg concentrations were measured with a total mercury analyzer (Model MERX, Brooks Rand) with cold vapor atomic fluorescence spectroscopy (CVAFS), according to the EPA 1631E Method.^29^ Elemental Hg was determined by directly purging a sample aliquot with high-purity N_2_ from a sealed 20 mL vial into CVAFS. As noted in Section 2.4, the total Hg (THg) was determined by dissolving the particulate Hg to Hg(II) with aqua regia and BrCl. An aliquot from the THg sample was transferred to a 20 mL vial prefilled with NH_2_OH·HCl to reduce excess BrCl, and then 100 μL of SnCl_2_ was added to reduce Hg(II) to Hg(0) followed by CVAFS analysis. In the case of field MEG, the dissolved Hg(II) was also measured. The field samples were first vigorously stirred to make sure the particles in the suspension were evenly dispersed. The aliquots were then taken for elemental Hg, dissolved Hg(II) (Method detailed in Text S3), and THg measurements. An *X*-point calibration was performed using a series of Hg(II) standard solutions (5–750 ppb, *R*^2^ > 0.999) with SnCl_2_ for Hg measurement.

### 2.6 DFT Computations to Assess the Effect of Solvent on the Thermal Decomposition Temperature of β-HgS

Density functional theory calculations were performed on [HgS_4_]^6–^ clusters by Gaussian 16 Revision C. 01.1.^30^ The original coordinates of the [HgS_4_]^6–^ clusters (HgS clusters for simplicity), Hg, and S are provided in Text S4. The B3LYP function^31^ is a cost-effective and accurate method to simulate electronic structures and estimate thermodynamic properties. The B3LYP function has been previously used to simulate mercury-bearing compounds.^32^ The energy-minimized structures were optimized without any strains using open-shell UB3LYP methods.^31^ The solvent effects were considered using the solvation model based on surface model density (SMD) with Stuttgart–Dresden basis set (SDD)^33^ for Hg, cc-pVTZ^34^ for S, and other lighter elements. The effective core potentials (ECP) of mercury were added to assess relativity effects.^33^ To evaluate the vibrational energy level of the optimized structures, the vibrational calculation was also performed using the same function and basis set as in Gaussian 16 Revision C. 01.1. The energy changes as a function of temperature were obtained via a linear regression. The molecular thermodynamic properties were analyzed by Shermo 2.6.^35^ The Hg–S bond dissociation energy regarded as the enthalpy changes (Δ*H*) of the reactions was also calculated.

## 3 Results and Discussion

### 3.1 Characterization of Synthesized β-HgS

**Figure 2:**
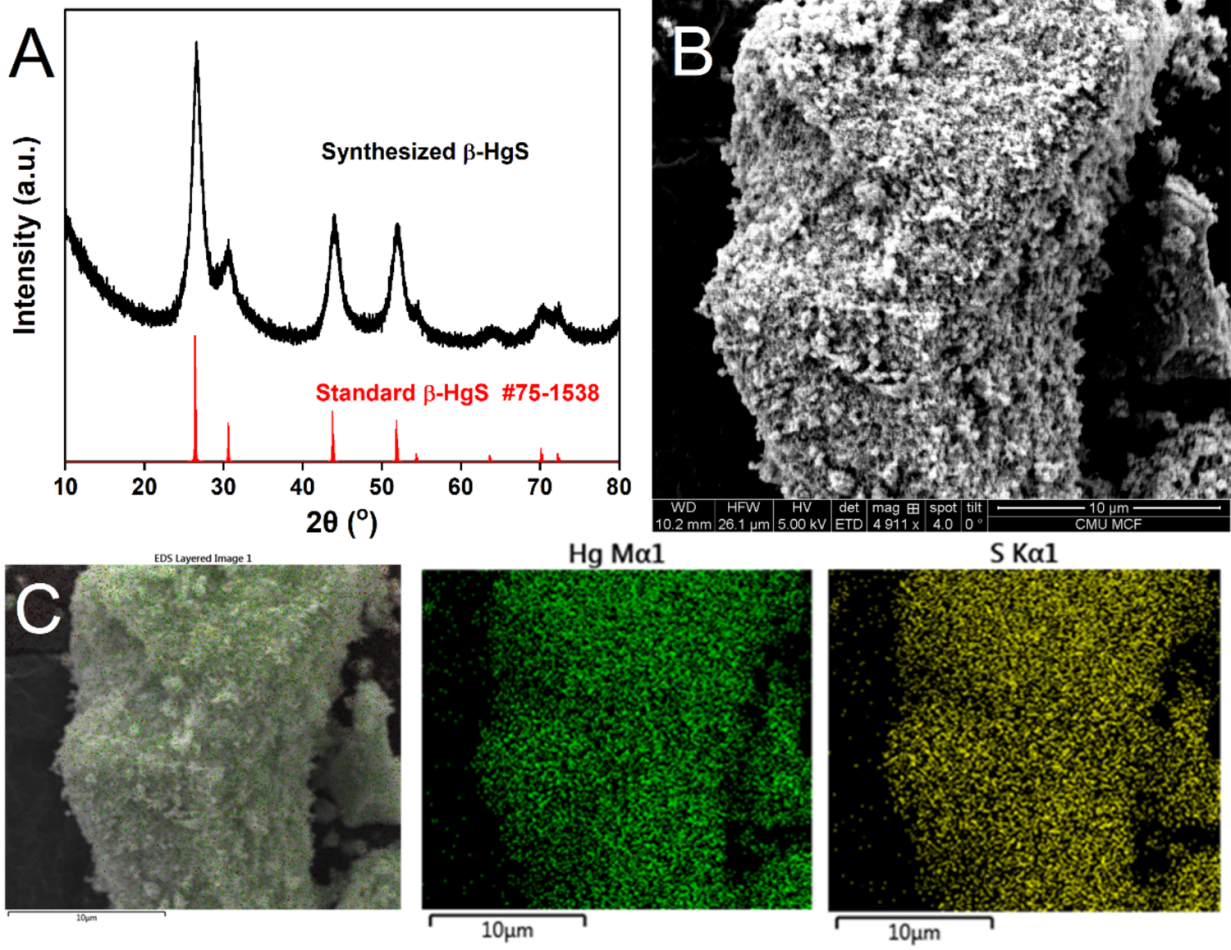
(A) XRD pattern of synthesized β-HgS. (B) SEM image of the synthesized β-HgS particles and (C) EDS for synthesized β-HgS showing the colocation of Hg and S in the particles.

The X-ray diffraction (XRD) pattern (Figure 2A) matches all of the standard peaks of β-HgS (metacinnabar) for Joint Committee on Powder Diffraction Standard (JCPDS) #75–1538.^36^ No additional peaks were observed, indicating the high purity of the prepared metacinnabar samples. The β-HgS XRD crystallite size was calculated to be 6 nm by using the Scherrer equation. A SEM image shows that the aggregated particles are composed of many smaller irregular particles (Figure 2B). The EDS analysis shows that the particles contain only Hg and S (Figure 2C). The relatively large particles observed in the SEM disaggregate when sonicated to form smaller particles as described later.

### 3.2 Characterization of Hg-Bearing Particles in Field MEG Samples

The total mercury (THg) concentrations in the two field MEG samples are 1412 ± 17 and 1658 ± 26 ppb, respectively. Whereas the Hg(0) + Hg(II)_aq_ concentrations in field MEG samples A and B are only 62 ± 1 and 34 ± 1 ppb, respectively (Table S2). Thus, approximately 95% of the Hg preset in field MEG existed as particulate species.

**Figure 3:**
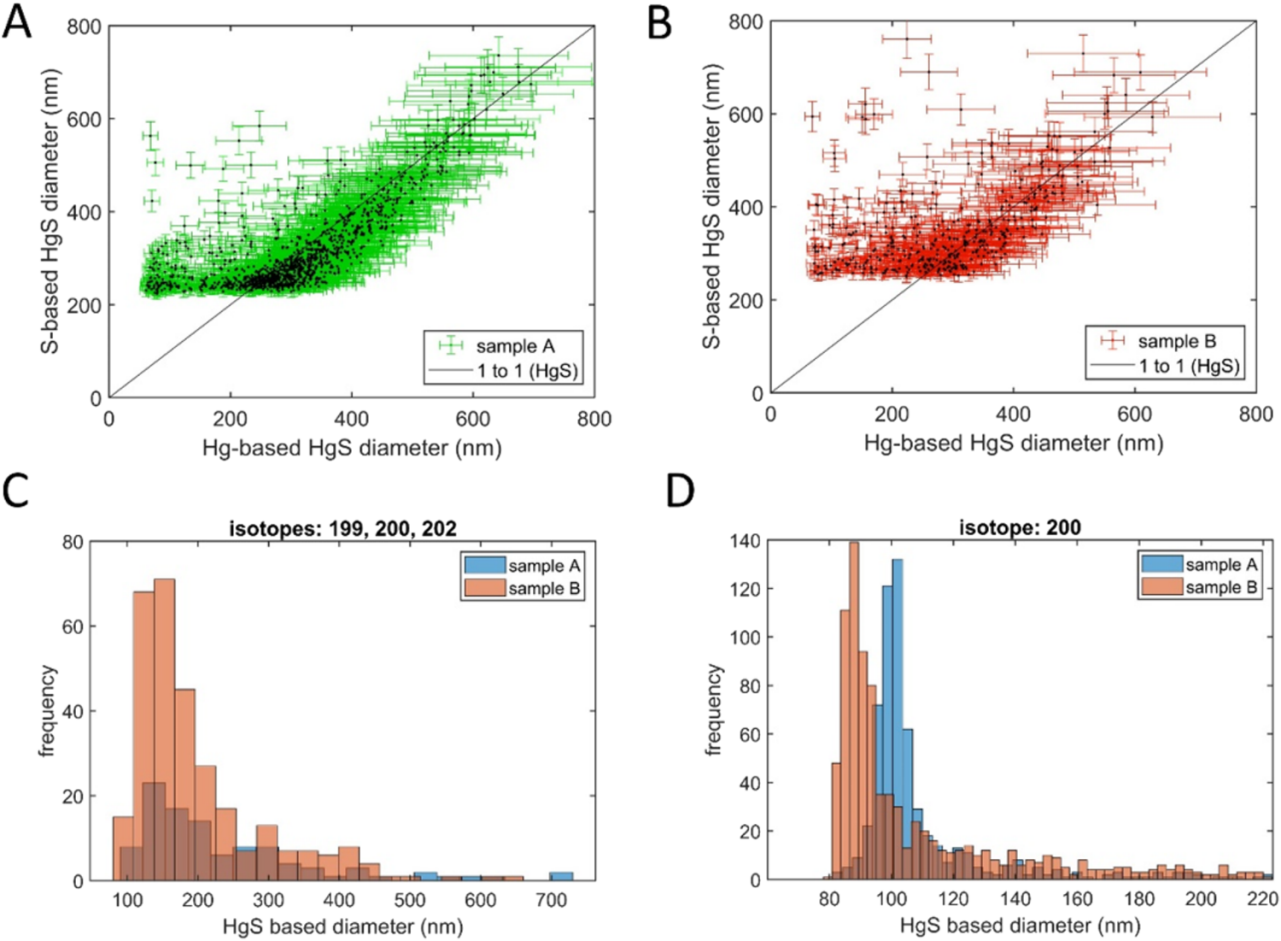
Relationship between S-based HgS diameter and Hg-based HgS diameter for feed MEG (A) sample A and (B) sample B. The size distributions of Hg particles in field MEG A and B based on (C) particles containing all three major Hg isotopes, and (D) containing only the most abundant Hg isotope (^200^Hg). The one-to-one line represents particles with 1:1 Hg:S stoichiometry found in β-HgS. The lower sensitivity of the spICP-TOF-MS for S compared to Hg means that the stoichiometry is confirmed only in HgS particles larger than about 200 nm.

To analyze the elemental composition of the Hg-bearing particles of field MEG, the particles filtered from field MEG were dispersed into water and analyzed using sp-ICP-TOF-MS.^28,37^ As shown in Figure 3AB, there are hundreds of individual particles in field MEG Samples A and B that contained both Hg and S. When the Hg-based HgS diameter was above 200 nm, most of the particles contained a Hg:S elemental ratio of 1:1, suggesting relatively pure HgS particles. When the HgS particle diameter was below 200 nm, the 1 to 1 Hg:S ratio disappears because the mass of S in the smaller particles is below the instrument detection limit. This is because low *m*/*z* elements like S (detected as ^32^S^16^O^+^*m*/*z* = 48) have a lower sensitivity on the ICP-TOF-MS compared to metallic elements like Hg (*m*/*z* > 200).^28^ The β-HgS particle size distributions were estimated in the samples based on the mass of ^200^Hg in the particle events and assuming the particles are HgS. The measured particles were centered around 100 nm for sample A and 90 nm for sample B (Figure 3D). If particle size distribution is determined only from the measured particles that contained all three mercury isotopes (^199^Hg, ^200^Hg, and ^202^Hg) to yield a higher certainty, the particles were in the range of 100–200 nm (Figure 3C). These results indicate that the Hg-bearing particles in field MEG samples are HgS with size ranging from 90–200 nm.

**Figure 4:**
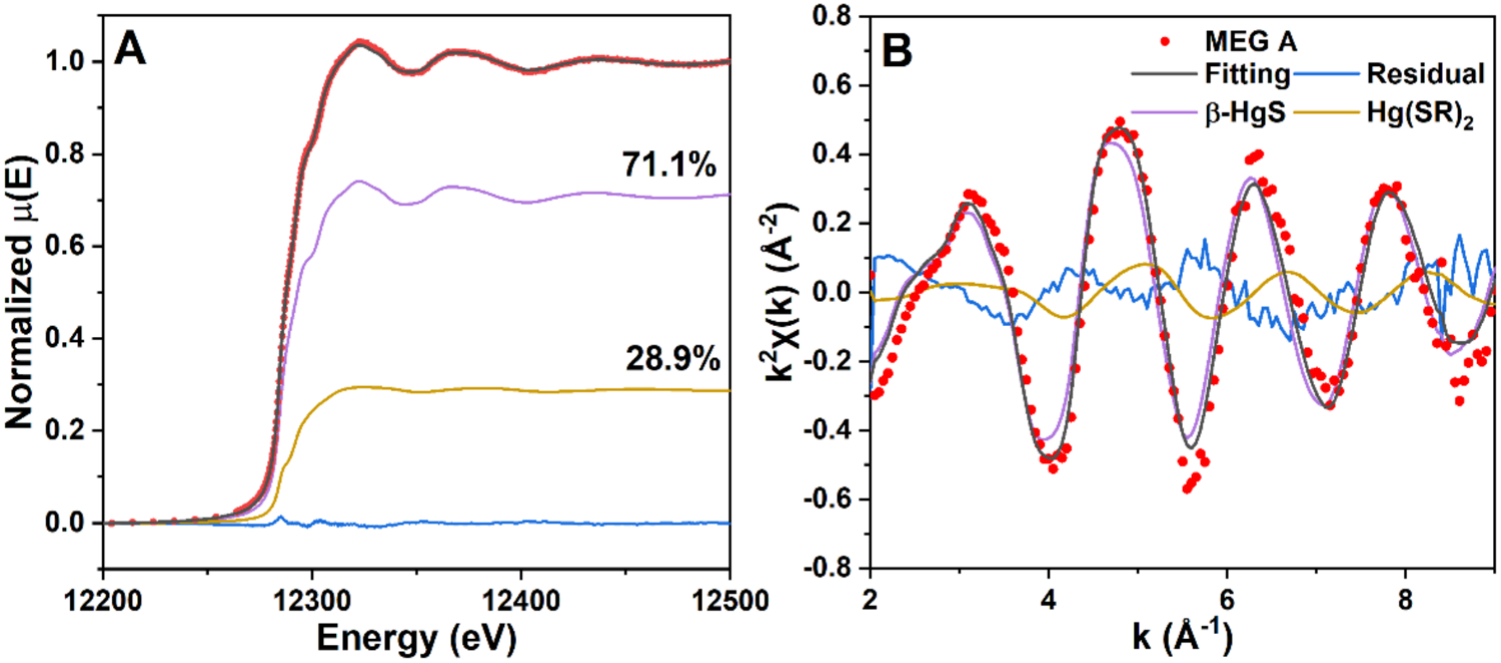
Hg L_III_-edge XAS spectrum of field MEG Sample A and the model compound spectra in (A) the XANES region and (B) the *k*^2^-space.

To confirm the Hg speciation in particles, XANES and EXAFS accompanied by linear combination fitting (LCF) were employed. Figure S2 shows the normalized Hg L_III_-edge XANES and EXAFS spectra of Sample A particles and model compounds. A characteristic peak at 12,323 eV in the XANES region and four peaks at 3.14, 4.80, 6.35, and 9.30 Å in the *k*^2^-space were observed in Sample A spectrum. A similar spectrum profile was also shown in two model compounds (β-HgS, Hg(SR)_2_), whereas other model compounds displayed different characteristic peaks (Figure S2). To quantify the percentage of different Hg species in the particles, LCF analysis with two components was applied to fit the Sample A spectrum with all the model compounds. The combination of β-HgS and Hg-thiol (Hg(SR)_2_) showed the best fit and the lowest residual in both XANES and *k*^2^-space (Figure 4). The fitting indicated that β-HgS is the major species (>70%) in Hg-bearing particles from field MEG samples (Table S3). This suggests that synthesized β-HgS should be a good surrogate for the Hg-bearing particles in field MEG in thermal decomposition experiments.

### 3.3 Synthesized β-HgS Thermal Decomposition

**Figure 5:**
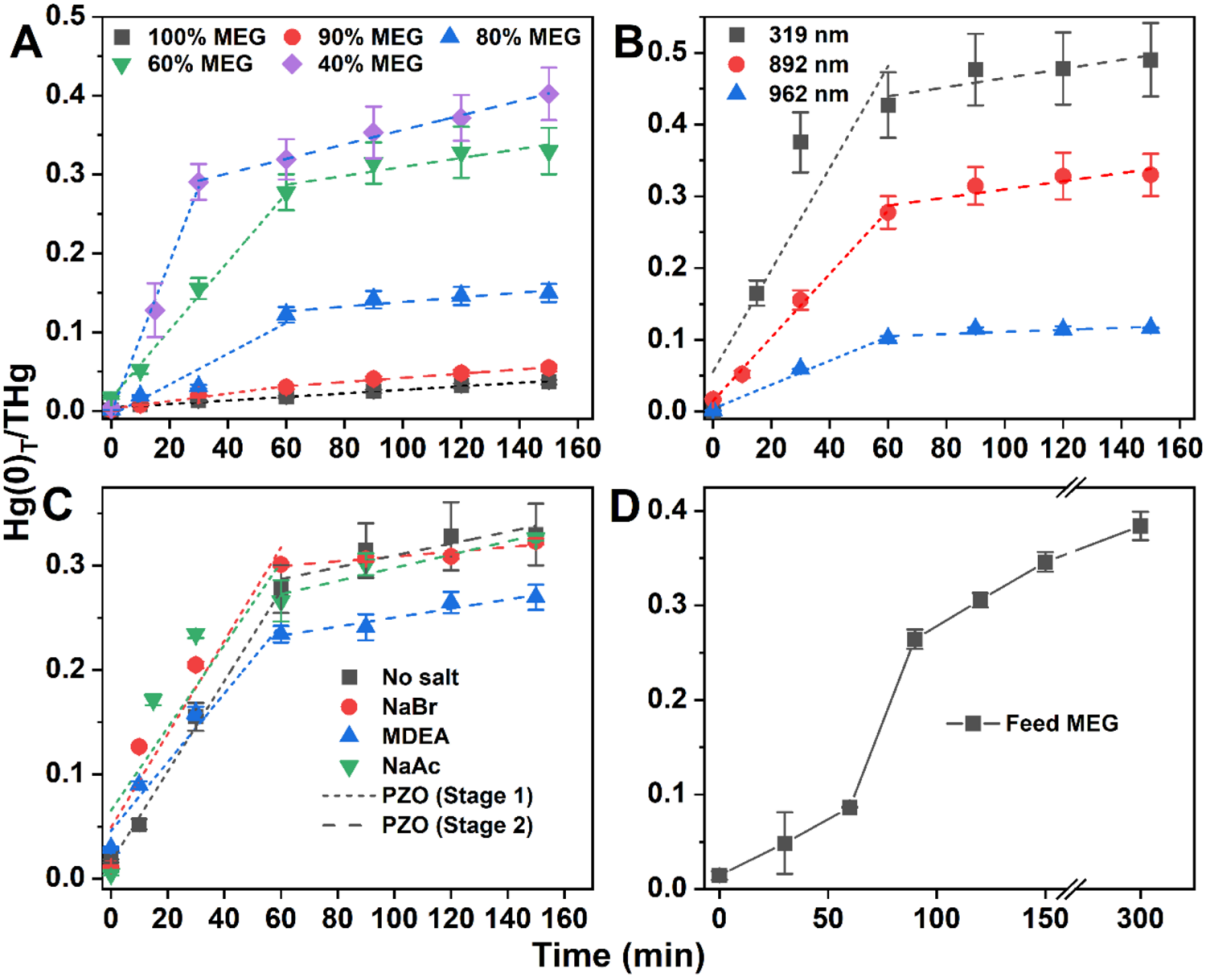
(A) Decomposition efficiency (Hg(0)_T_/THg ratio) of synthesized β-HgS at different MEG-water ratios during a 150 min heating period starting at *T* = 60 °C (*t* = 0). Dashed lines represent pseudozero-order fits for the time when water is present (initial rapid slope) and after the water has been evaporated (lower slope). The water is evaporated at approximately 60 min. The initial decomposition rate *k*_1_ increases from 0.22 × 10^–3^ to 9.55 × 10^–3^ min^–1^ with water content increasing from 0 to 60%, (B) Decomposition efficiency is shown for different β-HgS size distributions and (C) with and without coexisting constituents. (D) Decomposition efficiency of β-HgS in feed MEG sample A. Note that the N_2_ gas flow was maintained for an additional 150 min after the heating to make sure all of the Hg(0) in the water trap could be collected by the BrCl trap in the two-trap system.

Figure 5A–C and Table S4 summarize the measured β-HgS thermal decomposition profiles under the different conditions evaluated in this study. The THg mass balance ranged from 80 to 110% under all conditions (Table S4). Thermal decomposition of β-HgS was observed to start at approximately 100 °C. The percent of β-HgS mass that thermally decomposed to elemental Hg ranged from 3.7–49.0% at 130 °C, depending on the solution conditions and particle size (Figure 5A–C). In all cases, there was a rapid initial thermal decomposition rate (first 60 min) while water was present in the MEG, followed by a slower rate after 60 min when the water had boiled off to leave pure MEG. The curves were divided at 60 min and fitted by pseudozero-order kinetics separately (Table S5). The role of water content, particle size, and solutes on the rate of thermal decomposition are discussed in the next sections.

#### 3.3.1 Effect of MEG-Water Ratio

During the MEG regeneration process, i.e., distillation to remove water from rich MEG to reform lean MEG, there is a change in the MEG-to-water ratio. We explored the effect of the MEG-to-water ratio on the thermal decomposition of β-HgS (Figure 5A) by comparing the thermal decomposition of β-HgS in MEG to water ratios ranging from 100%:0% to 40%:60%. The initial β-HgS thermal decomposition rate increased with increasing water content in the reactor (Figures 5A and S2B). Once the water was boiled off, the rate of thermal decomposition slowed. For pure MEG and 90%:10% MEG:water, the rate was always slow. The decomposition efficiency of β-HgS at the end of the reaction was also positively correlated to water content (Figure S3A). These data indicate that the presence of water in MEG accelerates β-HgS decomposition. To confirm the accelerating effect of water, 72 mL of water (equivalent to 40% water content) was added back into a reactor that had been heated for 150 min after the water had boiled off. There was β-HgS still in the reactor. Upon reheating the reactor, the decomposition rate of the remaining β-HgS increased again while the water was present, and declined again after 60 min when the water boiled off (Figure S4). In a gas dehydration system, both rich and lean MEG always contain some water during the regeneration process,^38^ thus facilitating β-HgS thermal decomposition to elemental Hg during operation.

#### 3.3.2 Effect of β-HgS Size Distribution

The β-HgS particle size distributions also affected the rate of thermal decomposition to elemental Hg. As shown in Figure 5B, the thermal decomposition rate and efficiency are inversely proportional to the particle size distribution. About 43% of β-HgS with a 319 nm average hydrodynamic diameter (intensity weighted) was thermally decomposed in 60 min. In contrast, only 10% of β-HgS with a 962 nm average hydrodynamic diameter (intensity weighted) was decomposed in the same duration. Particles with an intermediate hydrodynamic diameter size range were between these values. The higher rate of thermal decomposition for smaller particles is likely due to their higher surface-to-volume ratio. After the water had evaporated off, the thermal decomposition rate slowed, but the β-HgS particles with the smallest size still had the highest decomposition rate in 100% MEG (0.635 × 10^–3^ min^–1^). The smaller particles have a larger surface-to-volume ratio, which provides a more reactive surface area for dissolution and promotes heat transfer with the surrounding medium, leading to more rapid thermal decomposition. The result from sp-ICP-TOFMS shows the size of HgS particles in field MEG is distributed in the range of 80–200 nm so the rates observed here for the smallest particles (319 nm average) may best represent the thermal decomposition rate expected for β-HgS in real field MEG. This is explored later in the paper.

#### 3.3.3 Effect of Coexisting Constituents

Unlike the synthesized β-HgS heated in pure water and MEG solutions, the field MEG contains many constituents, such as dissolved salts, hydrocarbons, and organic compounds, all of which may potentially influence the β-HgS thermal decomposition rate through interactions with the particles or products formed in the reaction. Figure 5C shows β-HgS decomposed in the MEG-water solution with common salts and compounds in field MEG. Compared to the pure solution (27.8% and 4.420 × 10^–3^ min^–1^), the addition of NaBr or sodium acetate (CH_3_COONa) did not have any significant effect on the decomposition efficiency or rate. MDEA is a common sweetening agent used to remove H_2_S and CO_2_ gas from crude oil and natural gas.^39,40^ The addition of MDEA only moderately (6%) decreased the decomposition efficiency (23.4%) and rate (3.290 × 10^–3^ min^–1^) compared to the pure solution. These results suggest that the coexisting constituents in the MEG-water solution have a minor influence on the observed β-HgS thermal decomposition. However, particle size and water content are key factors for controlling the thermal decomposition kinetics of β-HgS.

### 3.4 Thermal Decomposition of Hg-Bearing Particles in Field MEG

The thermal decomposition of β-HgS in field MEG shared a similar decomposition efficiency and profile as the synthesized β-HgS. As shown in Figure 5D and Table S6, for an initial Hg mass of 0.231 mg in the sample, the BrCl and water trap contained 38.4 and 5.9% of generated Hg(0) at the end of the reaction. Thus, 44.3% of Hg-bearing particles in field MEG have been thermally decomposed to elemental Hg at 130 °C. It should be noted that due to the presence of hydrocarbons in field MEG and the resulting high hydrocarbon gas flux leaving the reactor, 16.3% of the particulate β-HgS was transferred to the water trap without decomposition. Assuming this amount of β-HgS would have also decomposed if it had remained in the reactor, the thermal decomposition efficiency of field MEG would be higher. So, the thermal decomposition likely ranges from 44–60% of total particulate Hg in the field MEG. The size distribution of the Hg-bearing particles in field MEG (80 to 200 nm) is comparable to the synthesized 319 nm synthesized β-HgS. Approximately 49.0% of the 319 nm synthesized β-HgS was thermally decomposed to elemental Hg in a MEG-water system, which is consistent with the result from the field MEG. This suggests that the thermal decomposition of synthesized β-HgS provides a reasonable proxy for β-HgS in field MEG because the particle size distributions are similar.

### 3.5 Mechanism of HgS Thermal Decomposition in MEG-Water Solution

#### 3.5.1 Theoretical Calculation Results in Obtaining Thermodynamic Parameters

The frontier molecular orbitals of the [HgS_4_]^6–^ clusters with and without solvents are shown in Figure S5. As shown in Table 1 and Figure S6, the lower thermal decomposition temperature of β-HgS in water–MEG solutions than in the gas phase is supported by DFT calculations of the Hg–S bond enthalpy or bond dissociation energy (BDE) in gas, MEG, water, and a 40%:60% MEG:water mixture. A higher BDE indicates that more energy is needed to break Hg–S, which suggests a higher thermal decomposition temperature.

**Table 1:** Thermodynamic Parameters Calculated from Density Functional Theory (DFT)

	decomposition temperature (°C)	Δ*H* = BDE (kJ/mol)	Δ*S* (J/mol·K)	Δ*G* (kJ/mol)
no solvent	202.0	643.2	433.8	437.0
100% H_2_O	87.0	2.2	0.6	1.9
100% MEG	88.4	2.6	0.4	2.4
40%:60% MEG:H_2_O	89.1	2.7	0.4	2.5

In gas, the calculated Hg–S BDE varies slightly from 638.89 to 648.19 kJ mol^–1^ from 300 to 700 K. The solvent-free decomposition temperature calculated from BDE is 202.0 °C, which is consistent with the literature value.^20^ In solvents, the BDE is 2.16, 2.58, and 2.66 kJ mol^–1^ for 100% H_2_O, 100% MEG, and 40%:60% MEG:H_2_O, respectively. Based on these results, the solvents appear to dramatically decrease the enthalpy changes required for the thermal decomposition of β-HgS particles. The corresponding decomposition temperatures in these three solvents are calculated as 87.0, 88.4, and 89.1 °C. Other computed values, including Δ*S* and Δ*G*, are listed in Table 1. These decomposition temperatures are consistent with experimental observations of β-HgS thermal decomposition in MEG:H_2_O mixtures beginning at *T* = ∼90 °C. The relatively low rate of β-HgS thermal decomposition in pure MEG is inconsistent with these simulations. This suggests that the presence of water in the system affects the rate of thermal decomposition in an additional way aside from this thermodynamic solvent effect.

#### 3.5.2 Reaction Pathway of β-HgS to Elemental Hg

The redox reaction of β-HgS thermal decomposition in MEG is different from its decomposition in noble gases or hydrocarbons. To determine the reaction pathway, the synthesized β-HgS in 60%:40% MEG-water solution was thermally decomposed at 130 °C in a two-trap system (Figure 1B). The measured mercury and sulfur concentrations after decomposition are listed in Table S7. A total of 0.725 ± 0.004 mg (3.615 ± 0.016 μmol) Hg(0) was collected in the water and BrCl traps. That represents 16.2% decomposition β-HgS to form elemental Hg, consistent with the result in the one-trap system. Through ion chromatography analysis (Text S1), the dominant S species in the reactor and water trap after the heating is the sulfide ion, whereas sulfate is not detected. The sulfide mass is 0110 ± 0.003 mg (3.443 ± 0.018 μmol), which is nearly equimolar to the elemental Hg evolved in the reaction. This indicates that the thermal decomposition involves the dissolution of β–HgS to mercuric ion (Hg^2+^) and sulfide ion (S^2–^) in the MEG-water solution (eq 1). However, the released sulfide does not act as an electron donor to reduce the mercuric ion to elemental Hg, as is the case for β-HgS thermal decomposition in noble gas or hydrocarbons where the S(-II) is oxidized to S(0) and serves as the reductant for Hg(II) reduction to Hg^0^.^20,21^
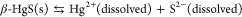
1

To determine the electron donor in the thermal decomposition reaction in MEG:water systems, HgCl_2_ was dissolved in 100% MEG and a 40%:60% MEG-water solution. As shown in Table S8, a significant amount of Hg(0) was produced in both the 100% MEG and 40%:60% MEG-water solution. Approximately 70.6 and 74.9% of the initial 1 mM Hg(II) concentration was reduced to elemental Hg, respectively, at 25 °C, indicating that Hg(II) is reduced by MEG, even at room temperature. The results indicate that MEG is the electron donor that reduces dissolved Hg(II) to Hg(0), likely oxidizing to an aldehyde or a carboxylic acid (eq 2). In comparison, hydrocarbons do not have hydroxyl groups. During thermal decomposition of β-HgS in hydrocarbons, the difference in molecular structures may cause electron transfer from S(-II) to Hg(II) and form Hg(0) and S_2_, as reported previously.^21^
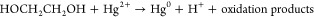
2

Even though the final MEG oxidation products (e.g., aldehydes or carboxylic acids) are unknown, it is evident that the redox between dissolved Hg(II) and MEG will be rapid at 130 °C and is not likely the rate limit step in β-HgS thermal decomposition. The dissolution of β-HgS to mercuric and sulfide ions is more likely to control the overall decomposition rate. Compared to MEG, water has stronger hydrogen bonding interaction toward β-HgS.^41^ Water may help to solubilize the Hg(II) released from β-HgS, which could explain the faster thermal decomposition rate when water is present compared to that with pure MEG.

## 4 Conclusions

Experimental measurements of β-HgS thermal decomposition confirmed that the solvent environment can significantly decrease the decomposition temperature. Water and MEG solvent environments decrease the decomposition temperature to <130 °C compared to >200 °C for the dry gas decomposition. The results also indicate that the presence of water in MEG and the β-HgS particle size distribution have a large effect on the thermal decomposition rate, while coexisting salts and organic solvents had little influence. The particulate Hg in field MEG (mostly β-HgS) displayed a similar thermal decomposition profile as the synthesized β-HgS with a similar size distribution, consistent with expectations based on these findings. DFT calculations accompanied by heating experiments revealed that the solvent (MEG/water) reduces the β-HgS bond dissociation energy, which leads to its thermal decomposition to mercuric and sulfide ions in MEG-water systems. The MEG acts as an electron donor to reduce the released Hg(II) to elemental Hg. The presence of water in MEG promoted the thermal decomposition reaction, likely helping to solubilize the Hg^2+^ ion released from the β-HgS particles. The results elucidate the reaction pathway for elemental Hg formation in the MEG regeneration process and determine the potential factors that may promote or hinder β-HgS decomposition in real operations.

## Supporting Information Available

Additional experimental methods; details of model compounds for XAS analysis and molecular orbitals for DFT calculation; the diagram of natural gas processing; and the mass balance and kinetic data of β-HgS thermal decomposition (PDF)
