# Supplementary material for: Thermal Decomposition of Metacinnabar (β-HgS) during Monoethylene Glycol Regeneration in Natural Gas Processing
Source: Energy Fuels. 2025 Apr 14;39(16):7841–9. doi: 10.1021/acs.energyfuels.5c00428 (PMC12035793; doi:10.1021/acs.energyfuels.5c00428)
Supplement: Supplementary file 1 — ef5c00428_si_001.pdf [file ef5c00428_si_001.pdf]

Supplementary Information (SI)

Thermal Decomposition of Metacinnabar (β-HgS) during Monoethylene Glycol Regeneration in Natural Gas Processing

*Chengyi Hong[1], Xiaopeng Huang[1], Tzu-An Lee[1], Yuanhao Zhou[1], Jonas Wielinski[1], Marcus Mello[2], Raja Jadhav[2], Daniel Chinn[2], Evan S. Hatakeyama[2], Thomas Hoelen[3], Gregory V. Lowry[1, *]*

[1]Department of Civil and Environmental Engineering, Carnegie Mellon University, Pittsburgh, Pennsylvania 15213, United States. [2]Chevron Technical Center, 100 Chevron Way, Richmond, CA 94802. [3]Chevron Oil, Products, and Gas, San Ramon, CA 94583.

Number of Text: 3

Number of Tables: 8

Number of Figures: 6

Text S1. Synthesized β-HgS thermal decomposition conducted in a two-trap system.

To reveal the mechanism of β-HgS decomposition, the two-trap system is used to capture elemental Hg and possible sulfur species produced from the decomposition. As shown in Fig. 1B, the system includes a stainless-steel reactor, a gas washing bottle filled with 50 mL of deionized water (Trap 1), and a graduated cylinder containing 100 mL BrCl solution (Trap 2). Seven mL of the β-HgS suspension was added to water-MEG (40%: 60%) solution in the reactor to reach the final volume of 180 mL. Starting at room temperature and 300 rpm of magnetic stirring, the reactor would gradually heat up to 130 ℃. After the reactor reached 60 ℃, the total heating and measurement time lasted 150 min. With continuous N_2 blowing through the headspace of the reactor, the produced elemental Hg would pass through the water trap and be collected by the BrCl trap. The water vapor and any sulfur species were transferred to the water trap bottle which is cooled by an ice-water bath. After the heating, the Hg(0) and Total Hg concentrations in the reactor and two traps were measured. The concentrations of different sulfur species in the reactor and water trap were identified and measured by ion chromatography (IC, IC-1000, Thermo Fisher, USA). The final liquid volume of the reactor and water trap were also measured to enable a mass balance calculation.

To determine the sulfur species after the decomposition, a series of sulfide (S^{2-}) and sulfate (SO_4^{2-}) standard solutions ranging from 0.1 ppm to 10 ppm were measured by IC to form standard curves. The solvents from Trap 1 and the reactor were first filtered by 0.22 µm membrane, and then the pH was adjusted to 9.5 – 10.5 by 1 M KOH. In this pH range, sulfide ions mainly existed in bisulfide (HS^-) form. After the adjustment, the samples were measured by IC to quantitively determine the concentrations of every sulfur species.

Text S2. Dissolved Hg(II) reduction reaction in different solvents.

To qualitatively determine whether mercuric ions (Hg^{2+}) can be reduced by monoethylene glycol (MEG), 1 mM $HgCl_2$ solutions were prepared in 100% MEG and water-MEG (40%: 60%) solvents. The solutions were poured into 40-mL vials, sealed, and shaken for one hour at room temperature. After shaking, the aliquots were taken for Hg(0) measurement.

Text S3. Measuring method of dissolved Hg(II) in field MEG samples.

The aliquots were taken from field MEG samples and transferred to a 20-mL vial. Then, 100 μL $SnCl_2$ solution was added to reduce dissolved Hg(II) to Hg(0). Finally, Hg(0) was determined by directly purging high-purity N_2 from the sealed 20-mL vial into the cold vapor atomic fluorescence spectroscopy (CVAFS). $SnCl_2$ solution can only reduce dissolved Hg(II) to Hg(0) without dissolving the particulate Hg species. Consequently, the Hg concentration measured using this method is comprised of any Hg(0) + $Hg(II)_{aq}$. The dissolved Hg(II) concentration is determined from the measured Hg(0) + $Hg(II)_{aq}$ minus the Hg(0) concentration.

Text S4. The original coordinates for HgS clusters, Hg and S for theoretical calculations.

The coordinates for HgS clusters.

Hg	-2.18804913	-0.51845342	0.00000000
S	-1.37136833	0.63647702	2.00041700
S	-4.63804913	-0.51842323	0.00000000
S	-1.37136833	0.63647702	-2.00041700
S	-1.37141049	-2.82834548	0.00000000

The coordinates for Hg.

Hg	-2.34622136	0.04393673	0.00000000

The coordinates for S.

S	-3.40070287	-0.43057996	0.00000000

Table S1. The origin and synthesis method of Hg model compounds used in XAS fitting.

Reference compound	Hg bond	Origin or synthesis method
$HgCl_2$	Hg-Cl	Purchased from Sigma-Aldrich
HgCl	Hg-Cl	Purchased from Sigma-Aldrich
Hg-phenyl	$C_6H_5HgOCOCH_3$	Purchased from Sigma-Aldrich
α-HgS	Hg-S	Method in [1]
β-HgS	Hg-S	Method in [1]
Hg-tetrathiolate	$Hg(SR)_4$	Method K in [2]
Hg-cysteine	$Hg(SR)_2$	Method A in [3]
Hg-cystine	$Hg(SR)_2$	Adapted from method A in [3]
Hg-thiosulfate	$Hg_2S_2O_3$	Method in [4]

Table S2. Hg(0), $Hg(II)_{aq}$ and THg concentrations of field MEG samples.

	Hg(0) (ppb)	$Hg(II)_{aq}$ (ppb)	THg (ppb)	$(Hg(0)+Hg(II)_{aq})$/THg ratio (%)
Sample A	16 ± 3	46 ± 3	1412 ± 17	4.4 ± 0.1
Sample B	8 ± 1	25 ± 1	1658 ± 26	2.0 ± 0.1

Table S3. The Hg speciation of Hg-bearing particles in field MEG Sample A based on linear combination fitting (LCF) results of XAS spectra using the model compounds in Table S1.

The spectrum assessed by LCF	Hg speciation (%)		Total (%)	R_f
	β-HgS	$Hg(SR)_2$		
XANES	71.1	28.9	100.0	1.42×10^{-4}
EXAFS	77.0	31.4	108.4	5.41×10^{-2}

Table S4. Mass balance calculation and Hg(0) detection of synthesized β-HgS thermal decomposition at different water-MEG ratio, particle size and co-existing constituents.

β-HgS decomposition conditions			Mass balance calculation				Hg(0) mass detected at selected intervals (ng)			
Average size (nm)	Water-MEG ratio	Co-existing constituents	Initial THg (mg)	THg in the trap (mg)	THg in the reactor (mg)	Recovery rate (%)	0 min	30 min	60 min	150 min
319	40%: 60%	No	0.265 ± 0.028	0.125 ± 0.005	0.109 ± 0.004	90.6 ± 9.5	4.8	23.7	22.3	5.8
892	40%: 60%	No	1.276 ± 0.100	0.421 ± 0.016	0.709 ± 0.002	88.6 ± 7.1	8.9	119.4	39.8	26.4
962	40%: 60%	No	6.037 ± 0.000	0.748 ± 0.043	4.603 ± 0.022	88.6 ± 0.8	29.2	500.0	220.7	42.8
892	0%: 100%	No	1.775 ± 0.062	0.065 ± 0.001	1.585 ± 0.018	93.0 ± 3.4	6.5	8.62	11.8	17.0
892	10%: 90%	No	1.775 ± 0.062	0.097 ± 0.002	1.355 ± 0.013	81.8 ± 3.0	8.5	11.9	9.6	11.7
892	20%: 80%	No	1.775 ± 0.062	0.266 ± 0.001	1.294 ± 0.004	87.9 ± 3.1	9.3	44.1	31.5	11.4
892	60%: 40%	No	1.775 ± 0.062	0.714 ± 0.021	0.897 ± 0.002	90.8 ± 3.4	8.0	259.0	119.2	8.2
892	40%: 60%	NaAc	1.372 ± 0.010	0.444 ± 0.002	0.715 ± 0.030	84.5 ± 2.3	3.4	316.6	59.2	24.2
892	40%: 60%	NaBr	1.372 ± 0.010	0.420 ± 0.001	0.878 ± 0.049	94.6 ± 3.6	2.3	403.9	26.0	1.54
892	40%: 60%	MDEA	1.333 ± 0.010	0.345 ± 0.010	1.112 ± 0.001	109.3 ± 1.1	2.8	382.8	33.7	42.2

Table S5. The parameters of pseudo-zero-order kinetic fitting for synthesized β-HgS thermal decomposition at early times in the presence of water (k_1) and later times after the water had boiled off (k_2).

β-HgS decomposition conditions			Stage one		Stage two	
Average size (nm)	Water-MEG ratio	Co-existed constituents	$k_1 \times 1000$ (min^{-1})	R^2	$k_2 \times 1000$ (min^{-1})	R^2
319	40%: 60%	No	7.090 ± 2.100	0.850	0.635 ± 0.240	0.778
892	40%: 60%	No	4.420 ± 0.169	0.997	0.567 ± 0.188	0.820
962	40%: 60%	No	1.670 ± 0.150	0.992	0.144 ± 0.070	0.682
892	0%: 100%	No	0.225 ± 0.014	0.982	--	--
892	10%: 90%	No	0.480 ± 0.051	0.979	0.267 ± 0.021	0.988
892	20%: 80%	No	1.950 ± 0.402	0.922	0.294 ± 0.086	0.854
892	60%: 40%	No	9.550 ± 0.750	0.994	0.922 ± 0.042	0.994
892	40%: 60%	NaAc	3.970 ± 1.570	0.760	0.632 ± 0.186	0.920
892	40%: 60%	NaBr	4.470 ± 0.878	0.928	0.227 ± 0.059	0.880
892	40%: 60%	MDEA	3.290 ± 0.400	0.971	0.433 ± 0.085	0.929

Table S6. Mass balance calculation for the thermal decomposition of field Sample A. Experimental conditions: 180 mL of field MEG sample, 130 ℃, 150 min of heating duration after the temperature reached 60 ℃.

Initial THg (mg)	Hg(0) in Trap 1 (mg)	Hg(0) in Trap 2 (mg)	THg in Trap 1 (mg)	THg in the reactor (mg)	THg(0)/ THg(initial) (%)	Recovery rate (%)
0.231 ± 0.001	0.014 ± 0.002	0.089 ± 0.003	0.051 ± 0.001	0.060 ± 0.011	44.0 ± 1.7	86.0 ± 4.9

Table S7. Mercury and sulfur mass balance calculation of synthesized β-HgS thermal decomposition in a two-trap system. Experimental conditions: 180 mL of 60%:40% MEG-water solution, 130 ℃, 150 min after the temperature reached 60 ℃.

	Hg(0) mass (mg)	THg mass (mg)	Dissolved sulfide mass (mg)
Reactor	0.000 ± 0.000	2.736 ± 0.094	0.033 ± 0.003
Trap 1	0.000 ± 0.000	0.291 ± 0.010	0.077 ± 0.000
Trap 2	0.725 ± 0.004	0.725 ± 0.004	--
Sum of the system	0.725 ± 0.004	3.753 ± 0.095	0.110 ± 0.003
Sum of the system (μmol)	3.615 ± 0.016	18.707 ± 1.768	3.443 ± 0.018
	THg(0)/ THg(initial) (%)	Hg mass recovery rate (%)	
Ratio	16.2 ± 0.2	83.8 ± 2.3	

* Initial THg added: 4.476 ± 0.053 mg

Table S8. Dissolved Hg(II) reduction reaction under different solvent conditions.

Solvent	Temperature (℃)	Initial Hg(II) concentration (ppm)	Final Hg(0) concentration (ppm)	Hg(0) conversion rate (%)
100% MEG	25	216.1 ± 4.4	169.8 ± 15.2	70.6 ± 15.7
60% MEG + 40% H_2O	25	226.6 ± 6.2	152.7 ± 33.7	74.9 ± 7.02

* Final Hg(0) concentration indicates the total Hg(0) concentration in reaction vials, including both dissolved and undissolved Hg(0).

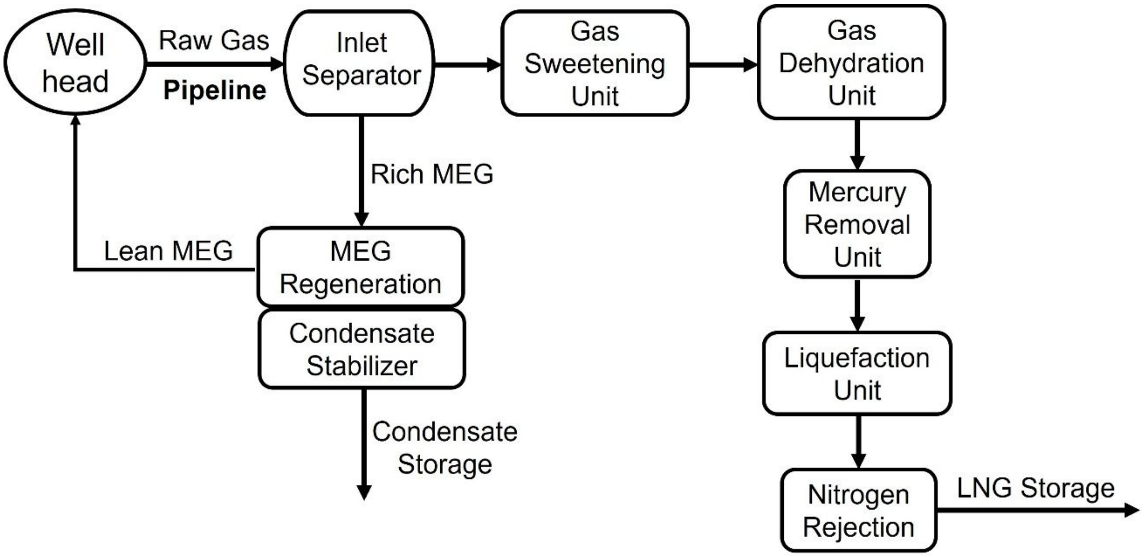

Fig. S1. The simplified flow diagram of natural gas processing systems.

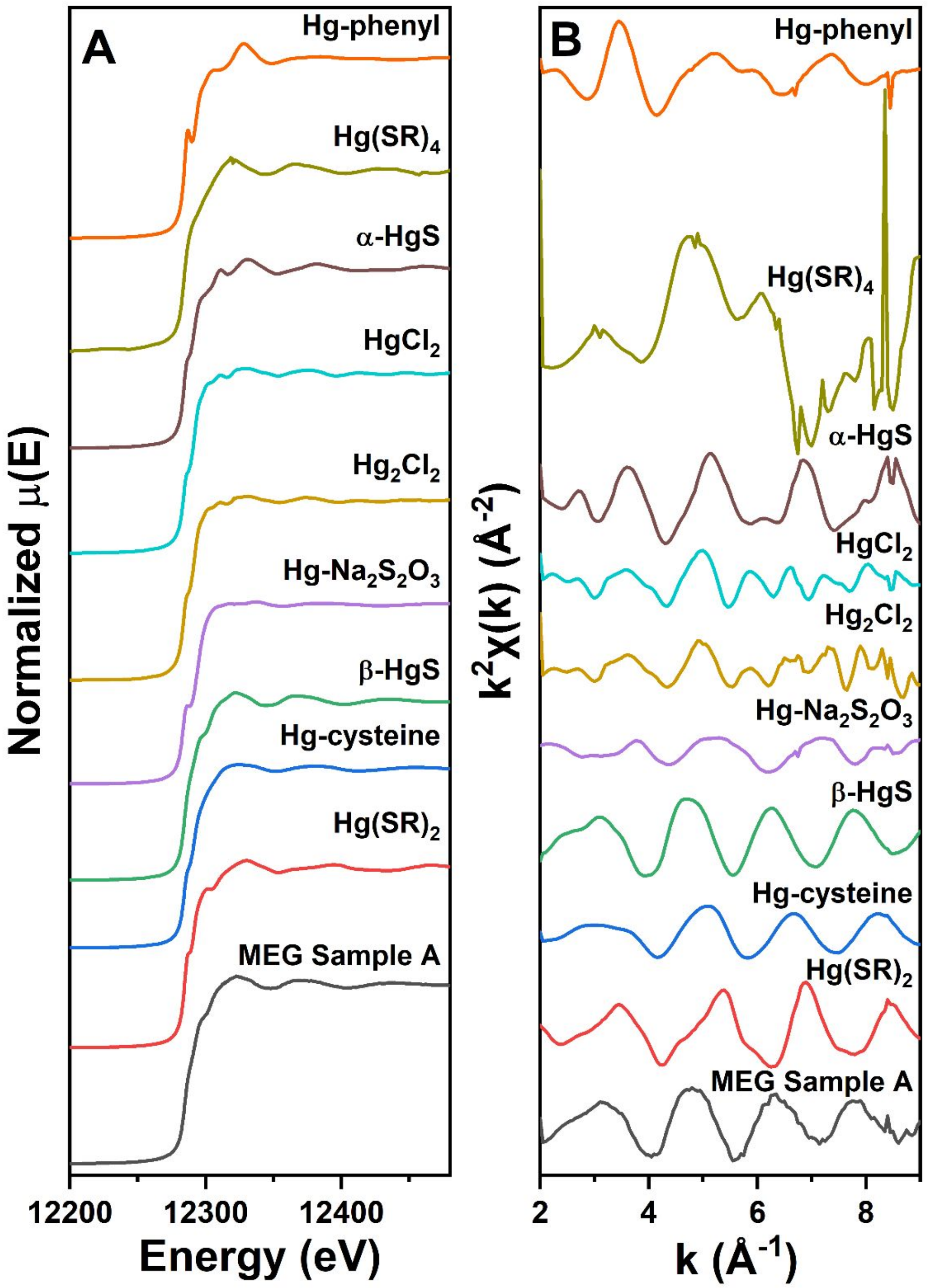

Fig. S2. The Hg L_{III}*-edge* XAS spectra of Hg-bearing particles in field MEG sample and the model compounds used for LCF analysis in (A) normalized XANES and (B) k^2-space of EXAFS region.

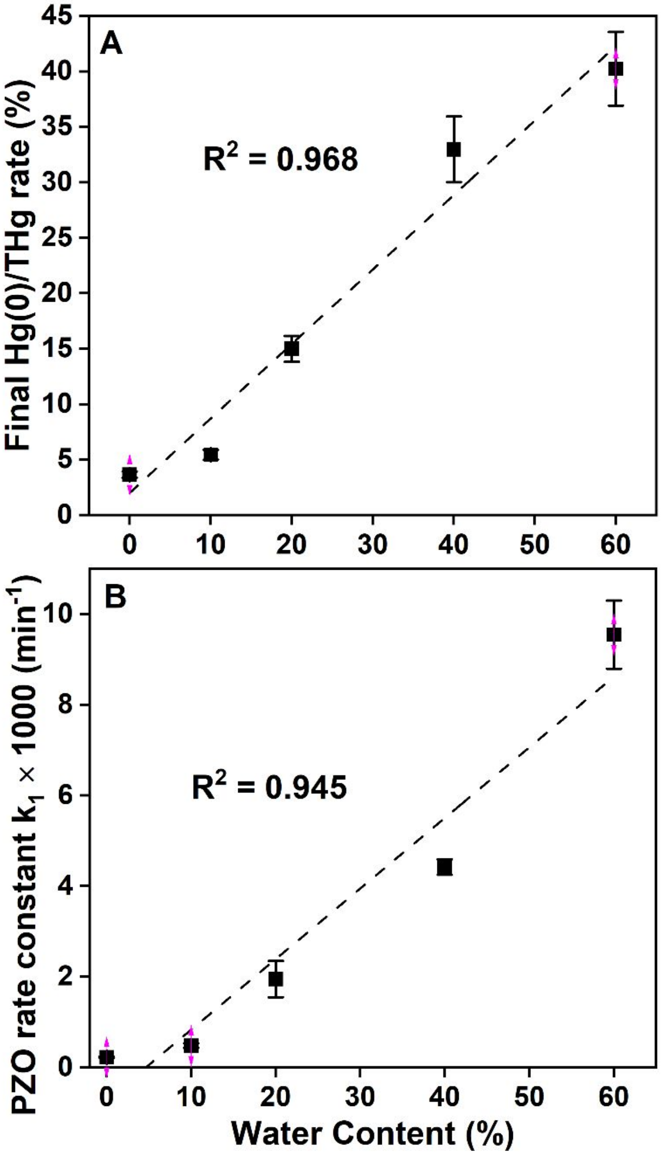

Fig. S3. The linear correlation between water content and (A) final decomposition efficiency (Hg(0)/THg), (B) Pseduo-zero-order (PZO) rate constant at stage one (k_1) during the thermal decomposition of synthesized β-HgS.

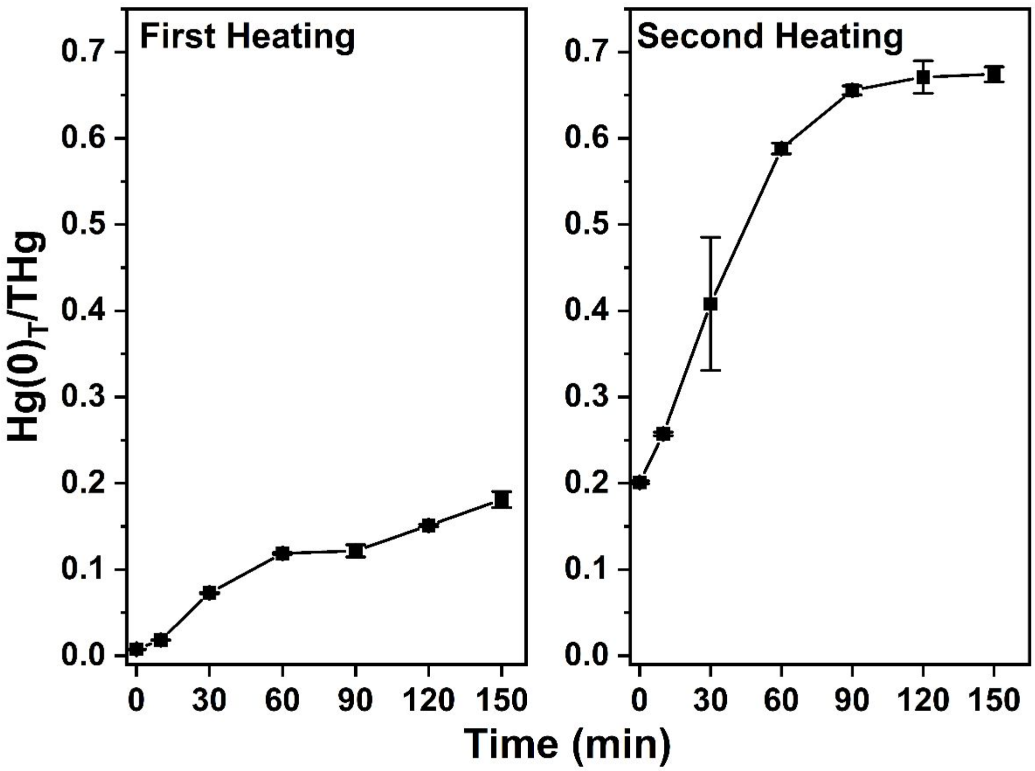

Fig. S4. The decomposition efficiency (Hg(0)/THg ratio) of synthesized β-HgS (892 nm) during two 150-min heating periods starting at 60 ℃ (t=0). The water is evaporated at approximately t=60 min. The initial water-MEG ratio is 40%:60%. After the first heating period, the reactor is cooled down to 60 ℃, 72 mL of water is added and reheated to 130 ℃ exactly as for the first period. The total decomposition efficiency is 67% after two heating periods.

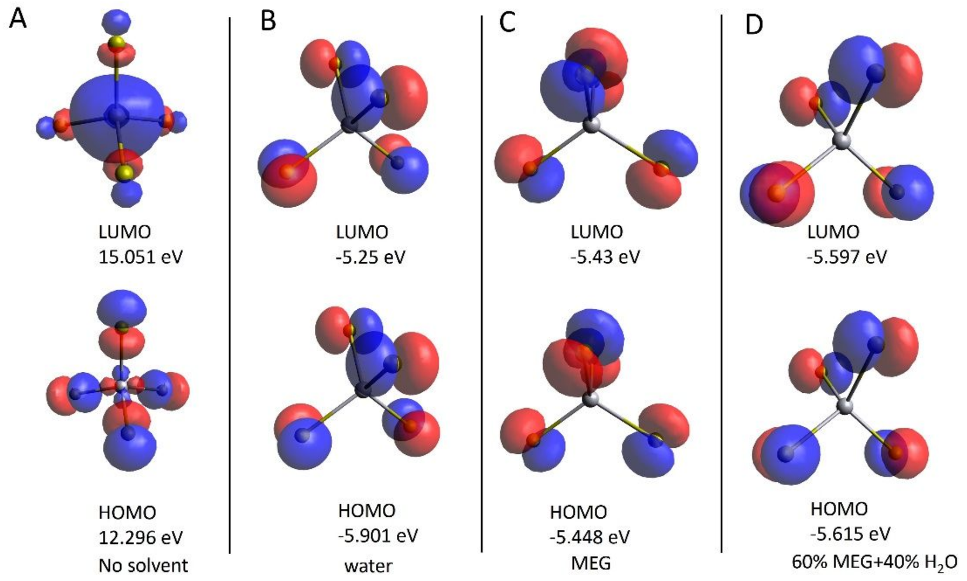

Fig. S5. The frontier molecular orbitals of the HgS clusters with and without solvents. UB3LYP, integral equation formalism polarizable continuum model (IEFPCM), SDD for Hg, cc-pVTZ for S and other lighter elements.

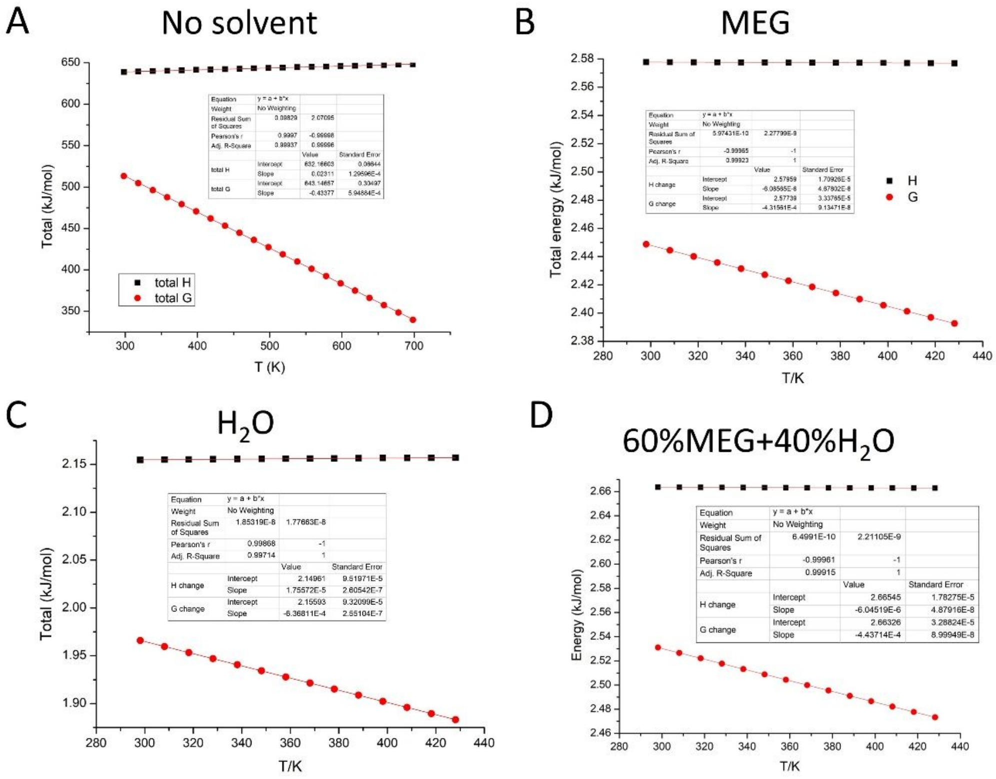

Fig. S6. Simulated thermodynamics parameters changes of β-HgS as a function of temperature at different solvent conditions.

Reference

1. Wang, H. and J.J. Zhu, *A sonochemical method for the selective synthesis of α-HgS and β-HgS nanoparticles.* Ultrasonics Sonochemistry, 2004. **11**(5): p. 293-300.
2. Warner, T. and F. Jalilehvand, *Formation of Hg(II) tetrathiolate complexes with cysteine at neutral pH.* Canadian Journal of Chemistry, 2016. **94**(4): p. 373-379.
3. Jalilehvand, F., et al., *Mercury(II) cysteine complexes in alkaline aqueous solution.* Inorganic Chemistry, 2006. **45**(1): p. 66-73.
4. Nyman, C.J. and T. Salazar, *Complex Ion Formation of Mercury(Ii) and Thiosulfate Ion.* Analytical Chemistry, 1961. **33**(11): p. 1467-&.
